# Muscle networks: Connectivity analysis of EMG activity during postural control

**DOI:** 10.1038/srep17830

**Published:** 2015-12-04

**Authors:** Tjeerd W. Boonstra, Alessander Danna-Dos-Santos, Hong-Bo Xie, Melvyn Roerdink, John F. Stins, Michael Breakspear

**Affiliations:** 1 MOVE Research Institute Amsterdam, VU University, Amsterdam, The Netherlands; 2 Black Dog Institute, University of New South Wales, Sydney, Australia; 3 School of Physical Therapy and Rehabilitation Science, University of Montana, Missoula, USA; 4 ARC Centre of Excellence for Mathematical and Statistical Frontiers, Queensland University of Technology, Brisbane, Australia; 5 QIMR Berghofer Medical Research Institute, Brisbane, Australia

## Abstract

Understanding the mechanisms that reduce the many degrees of freedom in the musculoskeletal system remains an outstanding challenge. Muscle synergies reduce the dimensionality and hence simplify the control problem. How this is achieved is not yet known. Here we use network theory to assess the coordination between multiple muscles and to elucidate the neural implementation of muscle synergies. We performed connectivity analysis of surface EMG from ten leg muscles to extract the muscle networks while human participants were standing upright in four different conditions. We observed widespread connectivity between muscles at multiple distinct frequency bands. The network topology differed significantly between frequencies and between conditions. These findings demonstrate how muscle networks can be used to investigate the neural circuitry of motor coordination. The presence of disparate muscle networks across frequencies suggests that the neuromuscular system is organized into a multiplex network allowing for parallel and hierarchical control structures.

Synchronous brain rhythms represent a dynamic mechanism for coordinating activity across large-scale neuronal networks and controlling the timing of neuronal firing^1^,^2^. Neuronal synchronization influences information flow through the central nervous system. Selective communication may be achieved through coherence of oscillatory firing rates between spatially disparate regions^3^. Although originally observed between pairs of neurons or neuronal ensembles^4^, the research on neuronal synchronization has been extended to investigate the organization of multiple brain regions into coherent brain networks^5^,^6^. Complex network analysis has been widely used to characterize the organization of distributed brain activity, revealing that brain networks share many features common to other complex physical and biological systems^7^. This approach has its origins in graph theory and describes complex systems by quantifying the topologies of their respective network representations^8^.

Neural synchrony has also been widely observed in the motor system where it may facilitate and increase the efficiency in controlling the execution of motor tasks^9^,^10^. For example, corticomuscular coherence has been observed between motor cortex and muscle activity^11^,^12^, and intermuscular coherence between different sets of muscles^13^,^14^. Equivalent to its role in perceptual processes, neuronal synchronization has been suggested to provide a mechanism for integrating the distributed motor and sensory systems involved in coordinated movement and posture^15^,^16^. As such, neuronal synchronization may provide a mechanism underlying the formation of muscle synergies^17^,^18^,^19^. The central nervous system has to deal with redundant degrees of freedom of the musculoskeletal system and muscle synergies may reduce this complexity through anatomical, mechanical or neural coupling between effectors^20^,^21^. Although this dimension reduction has been shown at the level of muscle activations^22^,^23^, its implementation in the central nervous system remains poorly understood.

Here we assess the topology of functional connectivity between ten leg muscles during various postural control tasks. If neural synchrony is a mechanism involved in the formation of muscle synergies, we expect a specific pattern of widespread connectivity between muscles that needs to be coordinated to maintain upright posture. Analogous to brain networks, we expect that these muscle networks will be task dependent and synchronize at multiple distinct frequency bands reflecting the spectral fingerprints of the neural circuitry involved. Apart from common input resulting from shared efferent and afferent pathways, we anticipate that spinal networks yield direct connections between motor-unit pools innervating different muscles. We estimate intermuscular coherence and partial directed coherence (PDC) between surface EMG to assess indirect and direct connections between motor-unit pools, respectively. We use graph theoretical analyses to quantify characteristics of the network topology and statistically compare muscle networks across four postural control tasks. In a previous study, we showed that these task variations had markedly different effects on postural dynamics^24^. If muscle networks are functionally organised, we expect different network topologies across task conditions. The topology and spectral profiles of muscle networks may provide novel insights into the neural implementation of muscle synergies. Muscle networks hence offer new tools to examine the neural circuitry involved in motor coordination.

## Results

We assessed intermuscular coherence between EMG envelopes of ten leg muscles – rectus femoris (RF), vastus medialis (VM), gastrocnemius (GM), tibialis anterior (TA), and extensor digitorum longus (ED) – while healthy adult participants were standing upright. Coherence has been widely used to investigate the linear coupling between neural activities^25^. Likewise, intermuscular coherence has been used to investigate oscillatory common inputs – here defined functionally as correlated inputs – of cortical or peripheral origin to different muscle groups^16^. Here we estimated intermuscular coherence between all muscle pairs (45 combinations) to define the edges of the undirected muscle network. We hence extended intermuscular coherence analysis to the multivariate domain and used complex network metrics to statistically compare the network topology across conditions. Muscle networks were assessed in four experimental conditions designed to induce subtle modulations of the basic standing pattern: (1) control, (2) counting backwards, (3) holding a cup, and (4) standing at height. We chose to assess EMG activity of the leg muscles during these task variations of quiet standing to avoid trivial changes in muscle networks resulting from muscles that become active or inactive in some task conditions.

**Figure 1:**
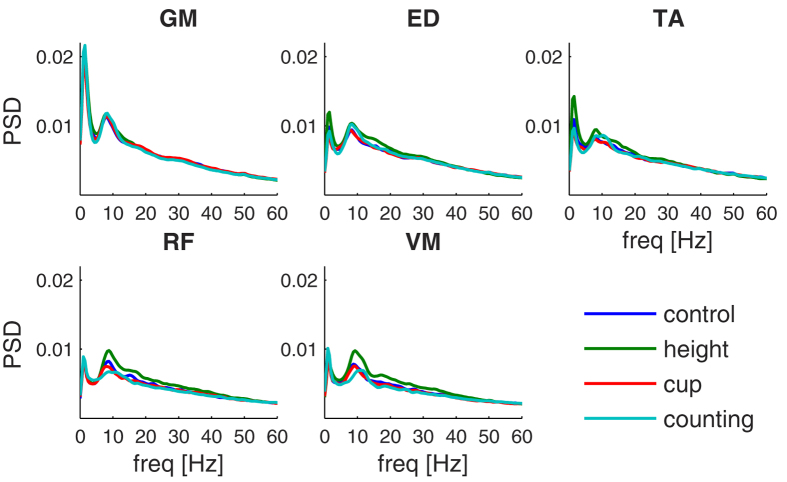
Normalized power spectral density (PSD) of the EMG envelope. PSD are shown for different muscles (GM: gastrocnemius, ED: extensor digitorum longus, TA: tibialis anterior, RF: rectus femoris, VM: vastus medialis) and in different conditions (blue = control, green = height, red = hold cup, cyan = counting). Power spectra were averaged across homologous muscles, trials and subjects and normalized to total power.

We first assessed the normalized power spectral densities (PSD) to investigate the spectral content of the EMG envelopes. PSD revealed a broad spectrum peaking around 9 Hz that was largely similar across muscles and conditions (Fig. 1). Visual inspection reveals that the main difference between conditions was a broadband increase in power in the *height* condition for the ED, TA, RF and VM muscles. The PSD in the other conditions were largely similar, although in the *counting* condition the peak at 9 Hz appeared more enhanced for the ED muscle and reduced for the RF and VM muscles.

### Undirected muscle networks

**Figure 2:**
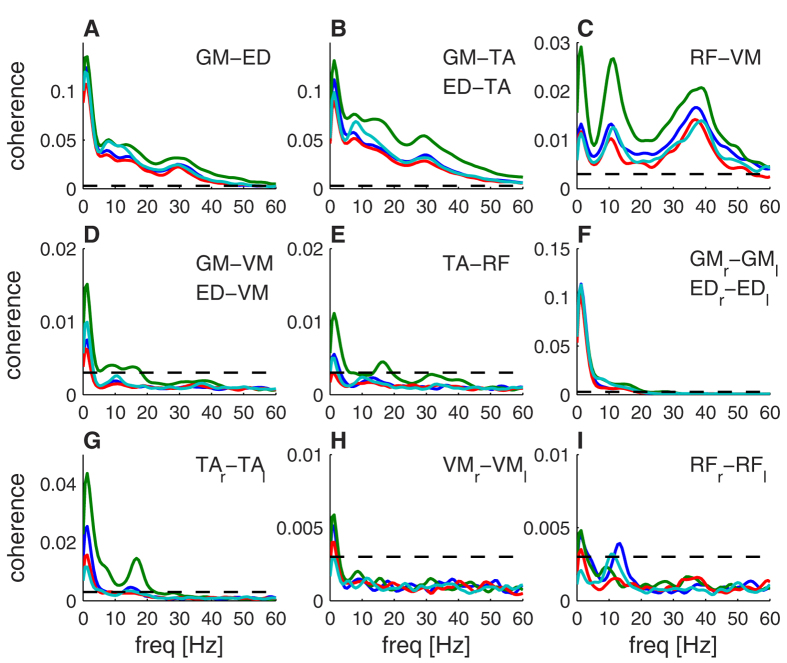
Intermuscular coherence for different conditions and muscle groups. (**A**) Between agonists in lower leg (GM-ED), (**B**) antagonists in lower leg (GM-TA, ED-TA), (**C**) antagonist in upper leg (RF-VM), **(D)** extensors in lower and upper leg (GM-VM, ED-VM), (**E**) flexors in lower and upper leg (TA-RF), (**F**) homologous extensors in lower leg (GM_r_-GM_l_, ED_r_-ED_l_), (**G**) homologous flexors in lower leg (TA_r_-TA_l_), (**H**) homologous flexor in lower leg (VM_r_-VM_l_), (**I**) homologous extensors in lower leg (RF_r_-RF_l_). Dashed lines show the 95% confidence intervals obtained through phase randomization of the EMG signals. Experimental conditions are reflected by line color (blue = control, green = height, red = cup, cyan = counting).

Intermuscular coherence differed between conditions and muscle combinations. In general, coherence was stronger for lower leg muscles and muscle combinations within the same leg segment. For instance, intermuscular coherence was significant for agonists (Fig. 2A) and antagonists (Fig. 2B) in the lower leg across a broad range of frequencies, with the strongest coherence at low frequencies. Intermuscular coherence was much reduced between antagonist muscles in the upper leg (Fig. 2C), but revealed pronounced peaks at 10 and 30–40 Hz. Coherence between lower and upper leg muscles (Fig. 2D,E) was weak with small peaks around 2, 10 and 16 Hz that crossed the 95% confidence interval (represented by dashed lines in Fig. 2). Intermuscular coherence between homologous muscles in lower left and right legs showed pronounced coherence at frequencies below 5 Hz and a secondary peak at 16 Hz (Fig. 2F,G). Coherence between homologous upper leg muscles was very weak (Fig. 2H,I), although a small peak around 10–16 Hz was observed between bilateral RF muscles (Fig. 2I).

**Figure 3:**
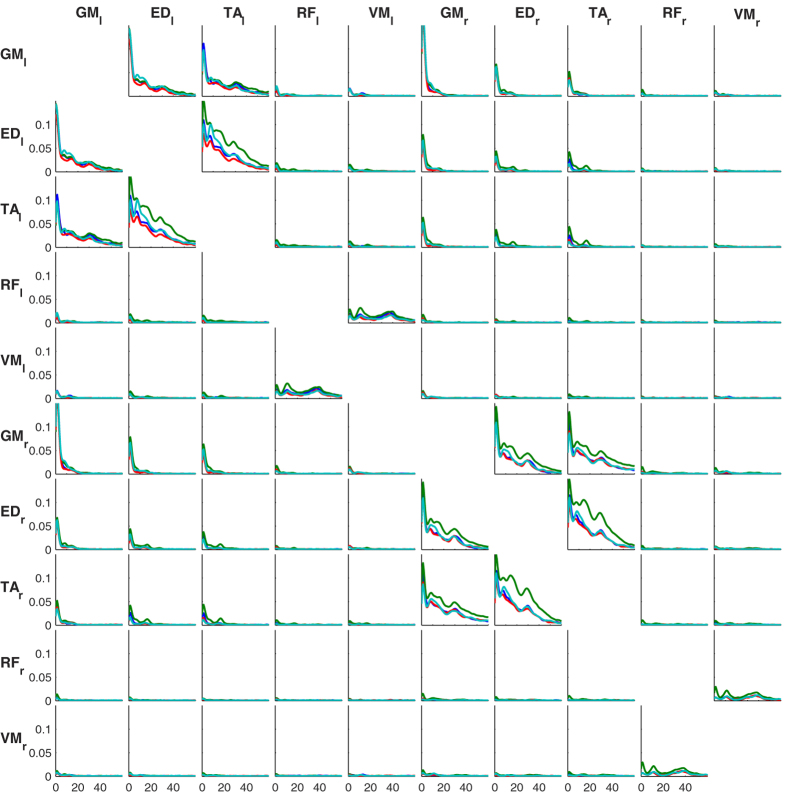
Intermuscular coherence between all muscle combinations. Coherence is shown between all 5 leg muscles (GM, ED, TA, RF and VM) on both sides (left indicated with a subscripted l, right with subscripted r) and for all conditions (blue = control, green = height, red = cup, cyan = counting).

Visual inspection suggests that intermuscular coherence is also dependent on the standing condition. In particular, higher coherence values in the *height* condition were observed for many muscle combinations and frequencies. During *counting,* increased coherence at 10 Hz was observed between agonists and antagonists in the lower leg (Fig. 2A,B). Compared to the *control* condition, intermuscular coherence appeared weaker when *holding a cup* for several muscle combinations, in particular between the upper leg muscles (Fig. 2C). The peak at 16 Hz was particularly pronounced between bilateral TA muscles in the *height* condition (0.014 ± 0.009), compared to the other conditions (control: 0.004 ± 0.002, cup: 0.002 ± 0.001, counting: 0.003 ± 0.002; ± indicates the standard error of the mean; Fig. 2G). Figure 3 shows intermuscular coherence between all muscle combinations, revealing a distinct functional organization of intermuscular coherence. Coherence is observed over a broad range of frequencies and the frequency profile depends on the muscle combination and the standing condition. The coherence shows a relatively sparse connectivity matrix with several muscle combinations showing little or no coherence.

**Figure 4:**
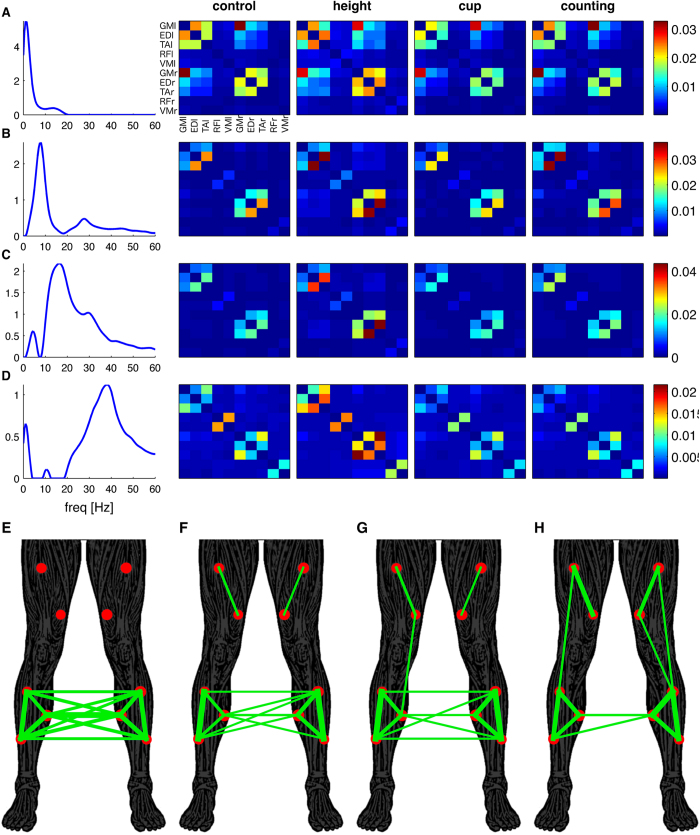
Undirected muscle networks obtained using non-negative matrix factorization of intermuscular coherence. The frequency content of the coherence spectra of all muscle combinations, conditions and subjects are decomposed into four components (**A–D**). Each factor is characterized by the extracted feature (frequency spectrum in the left column) and the loadings of this feature in original spectra. The right columns show the average loading across subjects for each condition separately (control, height, cup and counting). These loadings give the strength of the edges between the 10 nodes of each muscle network. Panels (**E–H**) show the binarised networks obtained using proportional thresholds (top 30%) for the networks corresponding to the frequency components in panels (**A–D**) respectively. The threshold was 0.0059, 0.0019, 0.0019 and 0.0014 for panels E-H, respectively. Connection strength is reflected by the width of the lines.

To assess muscle networks in distinct frequency ranges, we decomposed intermuscular coherence using a multivariate technique suited to strictly positive data sets, namely non-negative matrix factorization^26^. We extracted the common frequency components of the frequency spectra across all muscle pairs, conditions and subjects. Figure 4 shows four extracted components that explain most of the covariance and consist of the frequency component and the loadings in each muscle pair, condition and subject. The first factor captures coherence at very low frequencies (<5 Hz, Fig. 4A) and shows high loading between lower leg muscles within and across legs. The second factor shows a 6–10 Hz frequency component (Fig. 4B), the third factor a peak at 16 Hz (Fig. 4C) and the fourth component a broad peak between 30–45 Hz (Fig. 4D). These frequency components match the peaks observed in the coherence spectra in Fig. 2. The corresponding factor loadings yield the functional connectivity matrices of the muscle networks (same configuration as in Fig. 3) in each condition and subject. The bottom panels in Fig. 4 show the muscle networks in the *height* condition for each of the frequency components. These panels indicate a gradual transition from high bilateral connectivity between lower leg muscles for the lowest frequency component (Fig. 4E) to strong connections with upper leg muscles for higher frequency components accompanied by reduced inter-limb connections (Fig. 4H).

### Directed muscle networks

**Figure 5:**
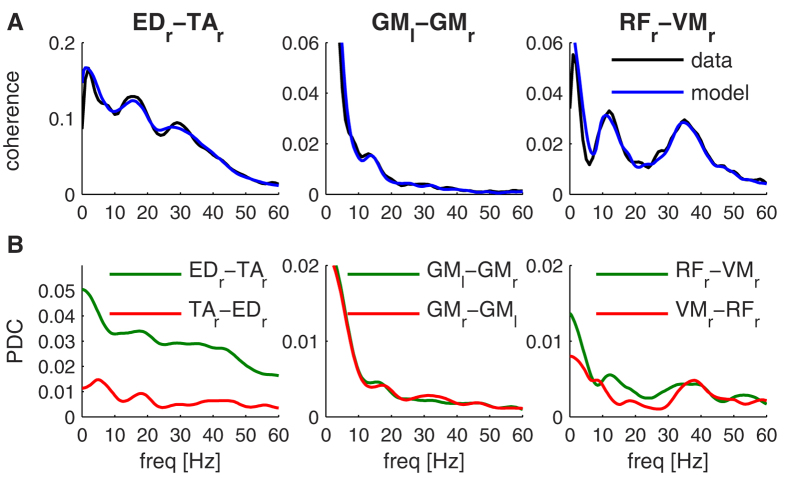
Coherence and partial directed coherence (PDC) obtained from MVAR model. (**A**) Comparison between coherence estimated from the empirical data using Welch method and coherence derived from the coefficients of the MVAR models that were fitted to the data. Intermuscular coherence between three muscle pairs are displayed (ED_r_-TA_r_, GM_l_-GM_r_, RF_r_-VM_r_); (**B**) PDC derived from the coefficients of the same MVAR models. In contrast to coherence estimates, PDC is a directed measure and connectivities in both directions are plotted.

Intermuscular coherence mainly reflects common inputs from other structures (indirect path) and therefore overestimates the strength of direct connections between motor-unit pools innervating different muscle muscles. This limitation of using linear correlations as a measure of network connectivity has been generally recognized^27^. Hence, in addition to intermuscular coherence, we also investigated partial directed coherence (PDC) as a measure of network connectivity. PDC takes into account the common input from other areas and is therefore a more direct reflection of coupling between two areas^9^. The optimal model order was determined for each condition and subject using Akaike’s information criterion and the average model order was 14.0 ± 3.7. In order to validate the model, we first computed magnitude-squared coherence from the model coefficients to compare against the coherence spectra estimated directly from the data. Figure 5A illustrates the coherence spectra for three muscle combinations that show strong intermuscular coherence, revealing a very good match between the spectra obtained from the model and the data. PDC was then computed from the same model coefficients to obtain directed muscle networks. PDC also revealed connectivity across a broad range of frequencies and muscle combinations but was generally weaker than coherence (Fig. 5B). PDC is a more stringent connectivity measure, which is less vulnerable to spurious contributions from indirect connections.

**Figure 6:**
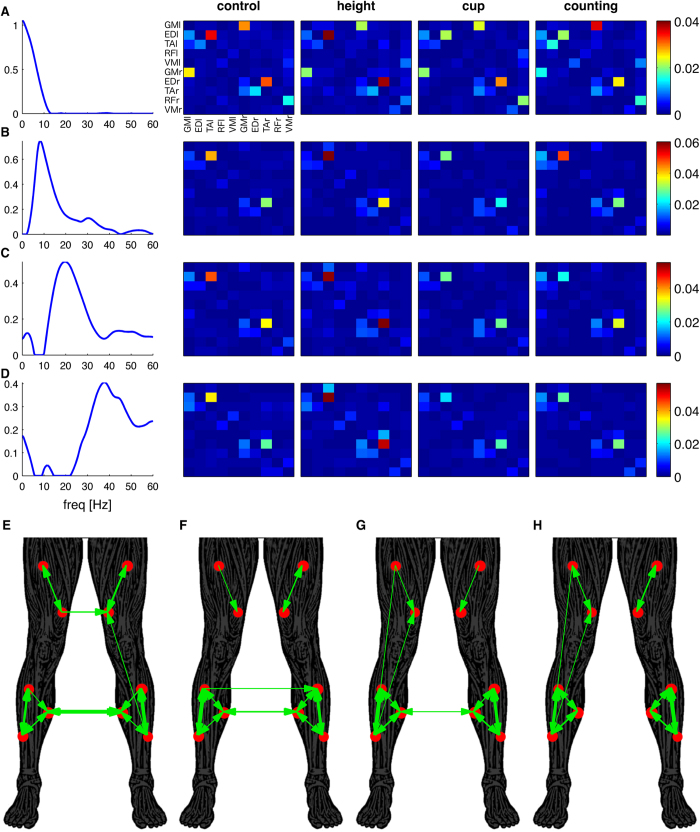
Directed muscle networks obtained using non-negative matrix factorization of PDC. The frequency content of the coherence spectra of all muscle combinations, conditions and subjects are decomposed into four components (**A–D**). Each factor is characterized by the frequency spectrum and the loadings of this feature in original spectra. The right columns show the average loading across subjects for each condition (control, height, cup and counting). Panels (**E–H**) show the binarised directed networks obtained using proportional thresholds (top 15%) for the networks corresponding to the frequency components in panels (**A–D**), respectively. The threshold was 0.0020, 0.0015, 0.0028 and 0.0018 for panels **E–H**, respectively. The arrows show the direction of connectivity. The width of the arrow reflects the connection strength.

Non-negative matrix factorization was again used to extract the common frequency components and obtain the corresponding connectivity matrices across conditions and subjects (Fig. 6). By definition, the connectivity matrices are positive as required for most complex network measures^8^. The resulting factors revealed similar frequency components as observed for intermuscular coherence, but the connectivity matrices were sparser. The first frequency component (<5 Hz) revealed strong connections between several muscle pairs (Fig. 6A), in particular between lower leg muscles as well as between bilateral GM muscles (Fig. 6E). The connectivity matrices of higher frequency components were sparser, showing strong connectivity from TA to ED muscle in both legs (Fig. 6B–D). Bilateral connectivity appears reduced at higher frequency components and largely confined to intra-limb connectivity at the highest frequency component, as reflected by the absence of bilateral connections in the thresholded network (Fig. 6H).

### Complex network analysis

**Figure 7:**
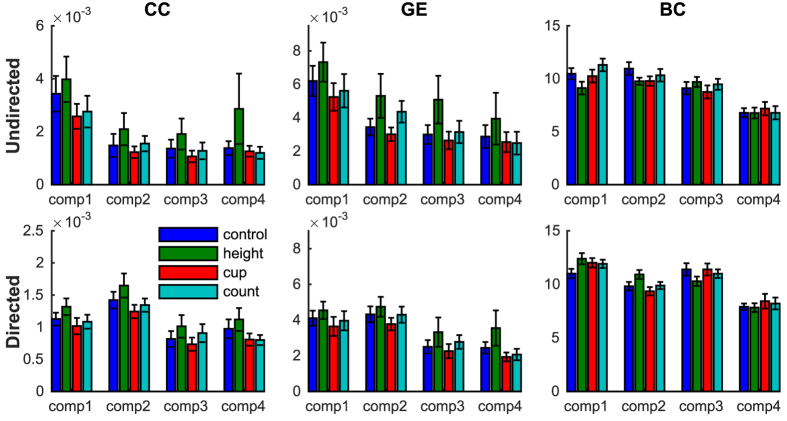
Summary statistics of complex network. Network metrics were used to statistically compare the muscle networks across conditions (blue = control, green = height, red = cup, cyan = counting) and frequencies (comp1 = 0–5 Hz, comp2 = 5–12 Hz, comp3 = 12–25 Hz, comp4 = 25–45 Hz). Clustering coefficient (**CC**), global efficiency (**GE**) and betweenness-centrality (**BC**) were assessed for the undirected networks obtained from intermuscular coherence (top row) and for the directed networks obtained from PDC (bottom row). Error bars reflect the standard error of the mean.

Complex network analysis was then used to compare the muscle networks between frequency components and conditions. The clustering coefficient (CC), global efficiency (GE) and betweenness-centrality (BC) were derived from both undirected and directed networks. Whereas the first of these three measures (CC) reflects network segregation, the latter two (GE and BC) are sensitive to integrative network properties. All three network measures showed significant main effects for frequency component in both the undirected and directed graph (*p* < 0.002), although the direction of the effect differed somewhat between network measures. Post-hoc t-tests revealed that for the undirected network (derived from intermuscular coherence) the CC and GE were the highest for the first frequency component (<5 Hz, Fig. 4A) compared to the other frequency components (CC: *p* < 0.005, GE: *p* < 0.01; Fig. 7, top row). For the directed networks, the CC was the highest for the second frequency component (~10 Hz, Fig. 6B) compared to the other frequency components (CC: *p* < 0.01; Fig. 7, bottom row). The GE was also the highest for the second frequency component, but pairwise t-test revealed that this was only significant compared to *comp3* (p = 0.003) and *comp4* (p < 0.0005). The BC decreased for higher frequency components in both the undirected and directed networks: the BC in *comp4* was significant lower compared to the lower frequency components (*p* < 0.0005; Fig. 6C). The low GE and BC for higher frequency components reflect the reduction in long-range connections between homologous muscles observed in the undirected (Fig. 4H) and directed (Fig. 6H) muscle networks.

The network measures also showed an effect of condition: the GE of the undirected network (*F*(1.2,21) = 4.7, *p* = 0.034), and the CC (*F*(3,51) = 3.8, *p* = 0.015) and GE (*F*(1.9,33) = 3.6, *p* = 0.045) of the directed network (Fig. 7). Post-hoc t-tests revealed that the CC was significantly higher for *height* compared to *cup* (*t*(17) = 2.9, *p* = 0.01) and for *height* compared to *counting* (*t*(17) = 2.5, *p* = 0.024) for the directed networks. The GE was significantly lower for *cup* compared to *control* (*t*(17) = 2.4, *p* = 0.027) and *height* (*t*(17) = 2.6, *p* = 0.02) for the undirected network, and compared to *height* (*t*(17) = 2.4, *p* = 0.029) for the directed network.

### Muscle synergies

**Figure 8:**
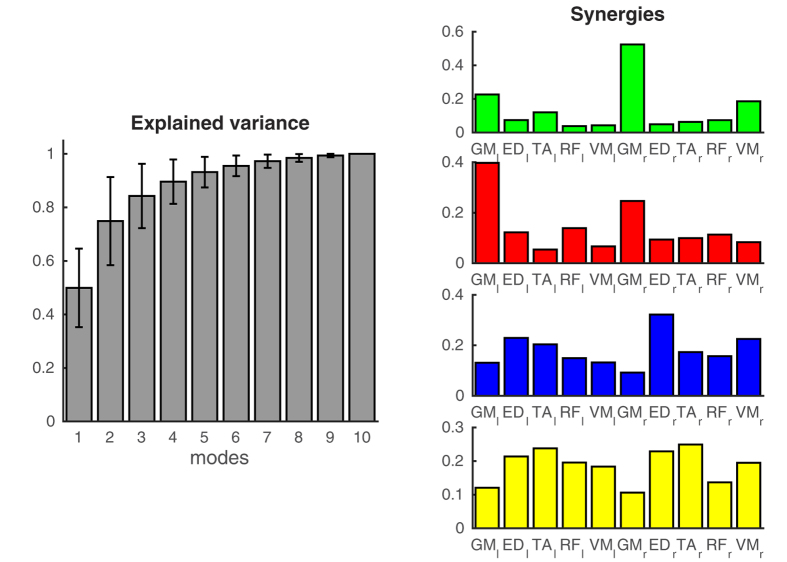
Muscle synergies extracted from EMG envelopes. The left panel shows the cumulative of the variance explained by the 10 principal components. On average, four components are required to explain 90% of the variance. The right panels show the four synergies extracted using non-negative matrix factorization.

Finally, we compared the current approach of mapping muscle networks to previous approaches that extract muscle synergies from the EMG envelopes. We thus decomposed EMG envelopes with non-negative matrix factorization and used principal component analysis to estimate the number of synergies. On average four principal components were needed to explain 90% of the variance. Four muscle synergies were extracted for each participant and condition separately and reordered as to maximize the correlation between synergies in different subjects and conditions. Figure 8 shows the average synergies that were extracted from the EMG envelopes. The first synergy (green) has a strong contribution of the right GM and additional contributions of the left GM, the right VM and the left TA. This synergy was most consistently observed in all subjects (*r* = 0.23 ± 0.11). 13 out of the 18 participants showed a particularly strong contribution from the right GM. The second synergy (red) has a strong contribution from the left GM and additional contributions of the right GM. The synergy was more variably observed across subjects (*r* = 0.10 ± 0.10) with 10 out of the 18 participants showing a particularly large contribution of the left GM. The third (blue) and fourth (yellow) muscle synergies have contributions of almost all the muscles and were rather variable across subjects (*r* < 0.06).

## Discussion

Motor synergies are crucial to human motor control, even during an apparently simple task such as standing upright. We here propose a novel approach referred to as muscle networks to gain new insights into the neural substrate of motor coordination. Muscle networks were reconstructed from EMG signals acquired from bilateral leg muscles during four different postural control tasks using a combination of time-frequency and multivariate analyses. Both undirected and directed connectivity was observed across a broad range of frequencies. Consistent muscle networks were observed across conditions and frequency components, although differences in network topology were evident: Lower frequency components show strong bilateral connectivity, whereas muscle networks at higher frequencies were largely confined to coupling within a leg. The clustering coefficient, global efficiency and betweenness-centrality differed significantly across frequencies, suggesting a multiplex network organization. Significant differences in network topology were also observed between experimental conditions, confirming that muscle networks are functionally organised. These findings show that muscle networks provide an innovative window into the neural implementation of muscle synergies. These findings also provide the foundation for future studies aiming to enhance the chances to detect abnormalities caused by neurophysiological changes that may lead to higher risks of falls in humans.

Muscle connectivity was observed at multiple distinct frequencies and, conversely, multiple connections between different muscle pairs were observed at each frequency. These findings extend previous work that has focused on intermuscular coherence between specific muscle pairs. Intermuscular coherence at very low frequencies (<5 Hz) is commonly observed during postural control and is thought to reflect the co-modulation of muscle activation^28^,^29^. Intermuscular coherence at higher frequencies has also been reported by several studies. We observed 6–10 Hz intermuscular coherence between leg muscles within and across legs^30^,^31^. Coherence at 16 Hz has also been observed between leg muscles^30^, in particular in patients with orthostatic tremor in which 16-Hz coherence is very pronounced^32^. Finally, intermuscular coherence at higher frequencies is less commonly observed, although a study on intramuscular coherence in the TA muscle did show coherence up to 45 Hz^33^, reporting a similar broadband coherence spectrum as that was reported here between lower leg muscles in the same leg (Fig. 2A,B).

We build on these pair-wise interrogations of intermuscular coherence through extensions to the multivariate domain, hence showing the presence of functional connectivity between groups of muscle at multiple distinct frequencies. Muscle connectivity at lower frequencies occurs over longer-range connections such as bilateral coupling between legs. In contrast, higher frequencies show shorter-range couplings (Fig. 4E–H). Intriguingly, this principle mirrors observations in cortical networks, whereby high frequencies characterize local coupling and lower frequencies typically couple over longer ranges – a pattern thought to pertain to cortical integration at different temporal and spatial scales^1^,^25^. Neural ensembles generally interact through multiple coupling mechanisms that have characteristic time scales or spectral signatures mixed together in the measures of network connectivity^34^. Network analyses of our data showed that muscle networks at higher frequencies were generally characterized by lower clustering coefficient, global efficiency and betweenness-centrality (Fig. 7), suggesting a distinct organization of muscle networks across frequencies. The multi-scale organization of cortical rhythms has been suggested to be vital for cortical information processing^35^, where different frequency bands process different aspects of incoming information – a phenomenon termed spectral processing^36^. Multiplex muscle networks may serve a similar function in motor control, such that synchronization in different frequency bands facilitate parallel and hierarchical control structures. The distinct topology of muscle networks across frequencies indicates that separate pathways underpin common input at different frequencies bands. By targeting specific frequencies, the underlying circuitry and functional role in postural control of these different frequency bands can be determined in future studies.

In addition to intermuscular coherence, we used partial directed coherence to quantify corresponding directed networks. PDC was observed in the same frequency bands, but was generally weaker than intermuscular coherence (Fig. 5). Robust patterns of PDC indicate the presence of direct neural coupling between muscles, as partial coherence distinguishes directed connections from two areas receiving a common input from a third area (see also^9^). Intermuscular coherence is generally thought to reflect common synaptic input arising from divergent supraspinal projections or afferent feedback^37^,^38^. The present findings suggest that intermuscular coherence is indeed largely generated by these common inputs, but in part also by spinal connections between motor-unit pools innervating different muscles. These findings are of interest in light of theories of inter-limb coordination, which mostly propose a contribution of spinal networks or central pattern generators (CPGs) to motor control^39^,^40^. CPGs refer to neural networks coordinating the activity of many muscles, but the mechanisms are only partly understood^41^,^42^. Because of the largely linear transfer function of a motor-unit pool^37^,^43^,^44^, the connectivity pattern between muscle activities can be used to infer the neural connectivity pattern among motor-unit pools^16^. Muscle networks may hence help to distinguish potential spinal network mechanisms, in particular when combined with computational neuromusculoskeletal models^45^,^46^,^47^. This combined approach has been particularly successful in delineating the mechanisms underlying cortical networks^7^,^48^.

We also evaluated muscle synergies extracted from EMG envelopes to compare our approach to existing methods used to investigate motor coordination. Several studies have used non-negative matrix factorization of the EMG amplitudes of multiple muscles to extract muscle synergies^22^,^49^,^50^. However, all studies involve dynamic movements or controlled perturbations of quiet standing^23^,^51^. In the present study – which involved quiet unperturbed standing – we did not find consistent muscle synergies across subjects. The first basis vector we extracted using non-negative matrix factorization was most consistent across participants, but in most subjects consisted mainly of the right GM muscle rather than a combination of muscles. The other basis vectors were rather variable across participants. We extracted four basis vectors for each participant and more consistent synergies may be extracted by using more advanced methods to estimate the number of synergies and match synergies across subjects^52^. However, it has been shown that when the number of synergies is close to the correct number, the features of the estimated synergies are preserved^49^. The inconsistent results reported here suggest that, in contrast to dynamic movements and perturbations, the EMG envelopes during quiet standing do not show stereotypical low-dimensional temporal patterns. The centre-of-pressure dynamics of postural sway are best characterized as a stochastic process without a well-defined temporal scale^53^,^54^. A corresponding absence of temporal scale in the EMG envelopes may underlie the absence of consistent muscle synergies during quiet standing. In contrast, the common input to the muscle as assessed using intermuscular coherence was characterized by specific frequency components that were consistent across subjects. By assessing correlated EMG activity in the frequency domain – in contrast to time domain analysis traditionally used to quantify muscle synergies – the current approach to assess muscle networks yields more robust results during quiet standing. Future work using dynamic tasks is required to more directly compare the present muscle network analysis to the widely used muscle synergy analysis.

In cortical systems, frequency-specific interactions likely reflect the neural circuitry that generates them with different circuitries generating different rhythms^55^. The same principles may hold for neuromuscular systems^16^. Neuronal synchronization has been suggested as a mechanism for integrating the distributed motor and sensory systems involved in coordinated movement and posture, i.e. the binding-by-synchronization hypothesis^15^. Muscle synergies represent a solution to the ‘inverse binding problem’ typical of sensory systems by encoding functional, task-relevant muscle coordination patterns^56^. The muscle networks observed in our study support this binding hypothesis, showing pervasive coupling among postural muscles. Multiple pathways converge onto the spinal motoneurons, Sherrington’s ‘final common path’ via which all motor commands must be relayed to the muscular apparatus. The observed muscle connectivity indicates that divergent projections are also an essential feature of the motor system. We here demonstrate how complex network analysis is ideally suited to investigate this many-to-many coupling in the motor system. This method may open new horizons to the detection of abnormal patterns of motor coordination resulting from progressive neurodegenerative diseases, lingering effects of CNS insults, and effects of medical interventions.

## Materials and Methods

### Participants

Eighteen healthy subjects participated (6 males, 12 females, mean age 26 ± 5 years) in the experiment. The study was approved by the ethics committee of the Faculty of Human Movement Sciences, VU University Amsterdam and performed in full compliance with the Declaration of Helsinki. All participants signed an informed consent form prior to testing.

### Procedure

We analysed EMG data from 10 leg muscles that was previously acquired during postural control under four different experimental conditions: (1) control, (2) counting backwards, (3) holding a cup, and (4) standing at height^24^. Holding a cup and saucer filled with liquid increases the total weight, but because the extra weight is relatively small in comparison to the total body weight, the effect on EMG and intermuscular coherence is likely negligible. Participants were invited to stand on a platform with their arms alongside the body. The experiment consisted of four blocks of four trials (each trial lasting 60 s) and conditions were presented in a pseudorandom order within each block. During the control condition, participants were standing quietly with their gaze fixated on the wall approximately 5 m in front of them. During the ‘counting backwards’ condition participants were counting backwards from a number (300, 301, 302, or 303) in steps of 7. In the ‘holding cup’ condition, participants were holding a cup and saucer filled with a cold dark liquid. In the ‘standing at height’ condition, the platform was raised to a height of 1 m. In all conditions participants stood with their toes nearly touching the edge of the force plate, so that in the height condition participants were facing a 1 m deep ‘cliff’ (for further details of the protocol, see^24^).

### Data acquisition

Bipolar EMG was recorded from 10 leg muscles using a 16-channel Porti System (TMSi, Enschede, The Netherlands). Electrodes were attached bilaterally to the muscle bellies of (1) rectus femoris (RF), (2) vastus medialis (VM), (3) gastrocnemius (GM), (4) tibialis anterior (TA), and (5) extensor digitorum longus (ED). To minimize ‘cross-talk’ between EMG signals, the inter-electrode distance between ED and TA electrode pairs was always greater than 2 cm. EMG was filtered online using a 5–400 Hz band-pass filter and digitized at 2 kHz.

### Connectivity analysis

To estimate intermuscular coherence, EMG signals were high-pass filtered (cut-off at 20 Hz) and rectified using Hilbert transform. The Hilbert amplitude yields the envelope of the broadband EMG signal and gives similar results as full-wave rectification^37^,^57^. Power spectral density and intermuscular coherence of the EMG envelopes was estimated using Welch method (window length 1 s, overlap 0.75 s). Complex-valued coherency was averaged across the four trials within each condition and squared to obtain magnitude-squared coherence. Intermuscular coherence was estimated between all 45 muscle pairs.

We utilized an extended MVAR model that combines both instantaneous and lagged effects (as implemented in the eMVAR Matlab Toolbox, http://www.science.unitn.it/~nollo/research/sigpro/eMVAR.html) to estimate two connectivity measures (coherence and partial directed coherence) from the coefficients of a MVAR model^58^. PDC provides a frequency-domain description of Granger causality, and can thus be used to estimate directed connectivity between network nodes^59^. To this end, the EMG envelope was band-pass filtered (0.5–70 Hz) and down-sampled to 200 Hz. All signals were mean-centered and normalized to unit variance to minimize the effect of differences in variances on the estimation of PDC^60^. A MVAR model was then fitted to the 10 EMG traces of each trial. We determined the optimal model order using Akaike’s information criterion^61^ for each trial separately. Coherence and PDC were derived from the fitted MVAR model coefficients^58^. As with intermuscular coherence estimated directly from the data using Welch method, the complex-valued connectivity measures (coherence and PDC) obtained from he MVAR model were averaged across trials and squared.

### Non-negative matrix factorization

Non-negative matrix factorization was used for spectral unmixing of the connectivity measures into distinct frequency components and their corresponding coupling strength. Non-negative matrix factorization is distinguished from other multivariate methods by its non-negativity constraints^26^. It has been applied to many problems including the extraction of time-varying muscle synergies^22^ and spectral unmixing for non-resolved space object characterization^62^. The connectivity measures were decomposed on the interval from 0–60 Hz into non-negative factors using an alternating least squares algorithm^62^. The spectra were hence decomposed into two non-negative matrices reflecting the spectral signatures (basis vectors) and the corresponding coupling strengths between all muscle pairs for each condition and subject, respectively.

### Complex network analysis

The connectivity strengths obtained through non-negative matrix factorization yield weighted matrices for network analysis. Subject-specific matrices were obtained, as we computed non-negative matrix factorization on the coherence spectra across conditions and subjects. For each adjacency matrix we derived three network measures: clustering coefficient, global efficiency and betweenness-centrality. All three network metrics were computed based on the weighted connectivity matrices^8^; muscle networks were only thresholded for visualization purposes. The clustering coefficient (CC) is a measure of functional segregation and is equivalent to the fraction of the node’s neighbours that are also neighbours of each other^63^. The mean CC for the network reflects, on average, the prevalence of clustered connectivity around individual nodes^8^, with higher values indicating a more functionally segregated network. The average shortest path length between all pairs of nodes in the network is known as the characteristic path length of the network^63^, which is the most commonly used measure of functional integration. The average inverse shortest path length is a related measure known as the global efficiency (GE). Higher values of GE indicate a more functionally integrated network. Paths length can be generalized for directed and weighted networks: weighted path length is equal to the total sum of individual link lengths, which are inversely related to edge weights^8^. Betweenness-centrality (BC) is a measure of centrality used to identify hubs in a network and is defined as the fraction of all shortest paths in the network that pass through a given node. BC is computed equivalently on weighted and directed networks, provided that path lengths are computed on respective weighted or directed paths.

All three measures were computed using the Brain Connectivity Toolbox (https://sites.google.com/site/bctnet) for both the undirected and directed networks. The CC and BC were averaged across nodes to obtain a global measure of the network^8^. We hence obtained a scalar value of the CC, GE and BC for all conditions, frequency components and participants. In order to test whether the muscle networks differed across conditions and frequency components, we performed a condition × frequency repeated-measures ANOVA on each connectivity measure for both the undirected and directed networks. If Mauchly’s test of sphericity failed to indicate normality, the Huyn-Feldt’s correction of degrees of freedom was used. Paired t-tests were used for post-hoc analysis to examine the differences among means when the F-test was significant.

### Muscle synergies

Muscle synergies were extracted using non-negative matrix factorization of the EMG envelopes^22^,^49^,^50^. Principal component analysis was used to estimate the number of synergies, determined by the number of principle components needed to explain 90% of the variance^23^,^51^. The EMG envelopes were hence decomposed into two non-negative matrices reflecting the synergies (basis vectors) and the corresponding activation patterns. Because muscle activation patterns were not constrained by a task or perturbation, muscle synergies were extracted for each participant and condition separately. We aligned the basis vectors by reordering them using a Procrustes analysis in order to maximize correlation across participants and conditions^6^.

## Additional Information

**How to cite this article**: Boonstra, T. W. *et al.* Muscle networks: Connectivity analysis of EMG activity during postural control. *Sci. Rep.* **5**, 17830; doi: 10.1038/srep17830 (2015).
